# The survival and clinicopathological differences between patients with stage IIIA and stage II rectal cancer: An analysis of 12,036 patients in the SEER database

**DOI:** 10.18632/oncotarget.12970

**Published:** 2016-10-28

**Authors:** Ben Huang, Shaobo Mo, Liang Zhu, Tianhong Xu, Guoxiang Cai

**Affiliations:** ^1^ Department of Colorectal Surgery, Fudan University Shanghai Cancer Center, Shanghai 20032, People's Republic of China

**Keywords:** rectal cancer, stage IIIA, stage II, survival

## Abstract

**Background:**

Stage IIIA rectal cancer has distinctive oncological features, including limited depth of intestinal wall invasion and early regional lymph node metastasis. We aim to compare survival outcomes and clinicopathological features for stage IIIA rectal cancer with those for stage II rectal cancer.

**Method:**

We analyzed patients with stage II or stage IIIA rectal cancer treated with surgery without receiving preoperative radiotherapy based on data from the US Surveillance, Epidemiology, and End Results (SEER) database between 1988 and 2003. Survival curves were plotted using the Kaplan-Meier method. Multivariate Cox proportional analyses were utilized to analyze independent prognostic factors for cancer-specific survival (CSS).

**Results:**

We included 12,036 rectal cancer patients (10,132 stage II and 1,904 stage IIIA) from the SEER database. Patients with stage IIIA rectal cancer had smaller tumor size than patients with stage II rectal cancer. A multivariate analysis suggested that compared with patients with stage IIIA rectal cancer, patients with stage II disease were more likely to have more unfavorable CSS (HR 1.195, 95% CI 1.079-1.324, p=0.001). When stage II rectal cancer was further analyzed as stage IIA, IIB and IIC rectal cancer, the multivariate analysis indicated that compared with patients with stage IIIA rectal cancer, patients with stage IIA rectal cancer (HR 1.113, 95% CI 1.003-1.235, p=0.044), stage IIB rectal cancer (HR 1.493, 95% CI 1.267-1.758, p<0.001) and stage IIC rectal cancer (HR 2.712, 95% CI 2.319-3.171, p<0.001) were also more likely to exhibit more unfavorable CSS.

**Conclusion:**

Patients with stage IIIA rectal cancer had more favorable survival outcomes and smaller tumor size compared with patients with stage II rectal cancer.

## INTRODUCTION

Rectal cancer is one of the most commonly diagnosed malignancies worldwide, with approximately 40 thousand new cases predicted to occur in the United States in 2016 [1]. The tumor, node, and metastasis (TNM) classification is currently the most frequently used rectal cancer staging system; it has been progressively updated to better tailor therapeutic strategies and predict oncologic outcomes [2–5]. In the 7th edition of the AJCC cancer staging system, tumors with lymph node metastasis, except for those related to metastatic rectal cancer, were divided into stage IIIA, IIIB and IIIC subgroups according to heterogeneous survival outcomes [6].

Stage IIIA rectal cancer is defined as tumors that invade submucosa with metastasis in 1-6 regional lymph nodes (T1N1–N2a) and muscularis propria with involvement of 1-3 regional lymph nodes (T2N1) [7]. Patients with stage IIIA rectal cancer were reported to have better survival outcomes compared with patients with other subgroups of stage III disease; the 5-year observed survival rates for stage III subcategories were 55% for stage IIIA, 35% for stage IIIB and 24% for stage IIIC [8]. Gunderson et al.[9] analyzed 35,829 patients from the SEER database and found that patients with T1-2N1/T1N2a rectal cancer had a 5-year observed survival rate comparable with patients with T2N0 disease (72%/73% vs. 75%, respectively). Li et al.[10] suggested that the 5-year overall survival of patients with stage IIIA colorectal cancer (86%) was greater than that of patients with stage II disease (75%), and comparable with that of patients with stage I disease (90%).

Multimodality therapy, i.e., preoperative chemoradiotherapy (CRT) followed by curative surgery, has been established as the standard treatment for patients with locally advanced rectal cancer during the last decade [11, 12]. Preoperative CRT followed by total mesorectal excision (TME) was reported to result in a decline in local recurrence and an improvement in oncologic outcomes in patients with cT3/T4 or node-positive rectal cancer [13]. Most patients respond to preoperative CRT, which downstages the tumor and leads to a considerable reduction in tumor burden, and almost 10%–30% of patients obtain a pathologic complete response (pCR) in their rectal cancer [14–16]. For patients with locally advanced rectal cancer with medical contraindication to multimodality therapy, CRT is also recommended as the standard adjuvant therapeutic strategy by National Institute of Health [17, 18].

Patients with stage IIIA colorectal cancer are reported to have a favorable prognosis; however, comparisons of survival outcomes and clinicopathological features between stage II and stage IIIA rectal cancer have rarely been performed. Here, we conduct an analysis that compares the survival and clinicopathological differences between patients with stage II and stage IIIA rectal cancer based on patient records in the US Surveillance, Epidemiology, and End Results (SEER) database.

## RESULTS

### Descriptive statistics

We included 12,036 rectal cancer patients (10,132 stage II and 1,904 stage IIIA) in the analysis. In total, 3,891 (32.3%) rectal cancer-specific deaths were identified. The median follow-up duration was 88 months (interquartile range, 36–126 months). Clinicopathological characteristics of the stage II and stage IIIA rectal cancer patients are shown in Table 1. The entire sample was comprised of 6,675 (55.5%) men and 5,361 (44.5%) women. The sample was predominantly Caucasian (84.3%), followed by African-American (7.0%). Histological types included adenocarcinoma (91.9%), mucinous cancer (7.8%), and signet-ring cell cancer (0.3%). Patients with stage II rectal cancer tended to be older than patients with stage IIIA disease (p<0.001). Cases with stage II rectal cancer had larger mean tumor size compared with cases with stage IIIA lesions.

**Table 1:** Demographics of patients with stage II and stage IIIA rectal cancer from the SEER database [N (%)]

Characteristics	Total	II	IIIA	P value
	(N=12036)	(N=10132)	(N=1904)	
Median follow-up (mos)	88	85	98	
Mean tumor size (cm)	4.8	5.0	3.5	
Sex				0.313
Male	6675(55.5)	5599(55.3)	1076(56.5)	
Female	5361(44.5)	4533(44.7)	828(43.5)	
Median age at diagnosis	69	69	65	
IQR	59-77	59-78	55-74	
Age at diagnosis (yrs)				<0.001
≤70	6674(55.5)	5421(53.5)	1253(65.8)	
>70	5362(44.5)	4711(46.5)	651(34.2)	
Race				0.030
White	10147(84.3)	8566(84.6)	1581(83.0)	
Black	838(7.0)	711(7.0)	127(6.7)	
Other ^a^	1051(8.7)	855(8.4)	196(10.3)	
Histological Type				0.296
Adenocarcinoma	11055(91.9)	9291(91.7)	1764(92.6)	
Mucinous adenocarcinoma	943(7.8)	810(8.0)	133(7.0)	
Signet-ring cell carcinoma	38(0.3)	31(0.3)	7(0.4)	
Grade of differentiation				<0.001
Well/Moderate	10425(86.6)	8851(87.4)	1574(82.7)	
Poor/Undifferentiated	1611(13.4)	1281(12.6)	330(17.3)	
Median LNH	8	8	8	
IQR	5-14	4-14	5-13	
LNH				0.009
<12	7938(66.0)	6633(65.5)	1305(68.5)	
≥12	4098(34.0)	3499(34.5)	599(31.5)	

LNH = number of lymph nodes harvested.

a Includes Native American, Asian, Pacific Islander and Unknown.

### Stage II vs. stage IIIA

**Figure 1:**
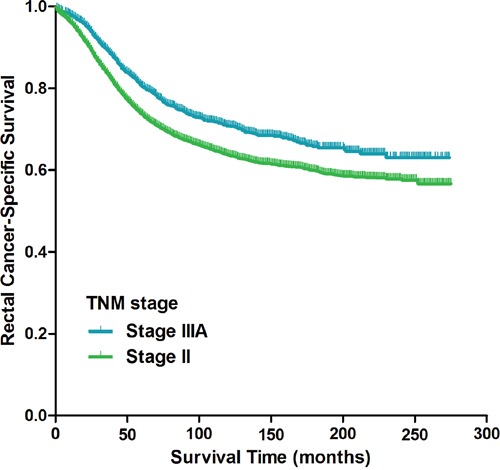
Kaplan-Meier curves of cancer-specific survival for patients with stage II and stage IIIA rectal cancer from the SEER database

Kaplan-Meier analysis suggested that patients with stage IIIA rectal cancer had a more favorable CSS compared with patients with stage II disease (p<0.001, Figure 1); the 5-year CSS of patients with stage IIIA and stage II rectal cancer was 80% (95% CI 80%-80%) and 73% (95% CI 72%-73%), respectively. Kaplan-Meier analyses of the entire cohort indicated that age at diagnosis (p<0.001), race (p<0.001), histological type (p<0.001), grade of differentiation (p<0.001), number of lymph nodes harvested (LNH) (p<0.001) and TNM stage (p<0.001) were risk factors associated with CSS (Table 2). Univariate Cox proportional hazard regression demonstrated factors associated with CSS were age at diagnosis (p<0.001), race (p<0.001), histological type (p<0.001), grade of differentiation (p<0.001), LNH (p<0.001) and TNM stage (p<0.001). An analysis using the multivariate Cox proportional model identified the following independent prognostic factors for CSS: age at diagnosis (p<0.001), race (p<0.001), histological type (p=0.009), grade of differentiation (p<0.001), tumor size (p<0.001), LNH (p<0.001), and TNM stage (p=0.001) (Table 3). Compared with patients with stage IIIA rectal cancer, patients with stage II disease were more likely to have more unfavorable CSS (HR 1.195, 95% CI 1.079-1.324, p=0.001). Kaplan-Meier curve of overall survival for patients with stage II and stage IIIA rectal cancer was shown in Figure S1 (p<0.001).

**Table 2:** Univariate survival analyses of patients with stage II and stage IIIA rectal cancer from the SEER database

Variable	No.	5-year CSS	Log rank χ^2^	P-value
Sex			1.545	0.214
Male	6675	74%		
Female	5361	74%		
Age at diagnosis (yrs)			294.925	<0.001
≤70	3494	79%		
>70	8542	66%		
Race			57.126	<0.001
White	10147	74%		
Black	838	65%		
Other ^a^	1051	80%		
Histological Type			32.868	<0.001
Adenocarcinoma	11055	75%		
Mucinous adenocarcinoma	943	66%		
Signet-ring cell carcinoma	38	54%		
Grade of differentiation			14.664	<0.001
Well/Moderate	10425	75%		
Poor/Undifferentiated	1611	69%		
LNH			95.038	<0.001
<12	7938	71%		
≥12	4098	80%		
TNM stage			41.709	<0.001
IIIA	1904	80%		
II	10132	73%		
TNM stage ^b^			364.717	<0.001
IIIA	1904	80%		
IIA	8852	75%		
IIB	693	67%		
IIC	587	46%		

CSS = cancer-specific survival, LNH = number of lymph nodes harvested.

a Includes Native American, Asian, Pacific Islander and Unknown.

b Stage II rectal cancer was analyzed as Stage IIA, IIB and IIC rectal cancer.

**Table 3:** Multivariate survival analyses of patients with stage II and stage IIIA rectal cancer from the SEER database

Variable	Multivariate analysis		P value
	HR	95% CI	
Age at diagnosis (yrs)			<0.001
≤70	1	reference	
>70	1.686	1.572-1.807	
Race			<0.001
White	1	reference	
Black	1.450	1.283-1.639	<0.001
Other ^a^	0.767	0.671-0.877	<0.001
Histological Type			0.009
Adenocarcinoma	1	reference	
Mucinous adenocarcinoma	1.195	1.054-1.355	0.006
Signet-ring cell carcinoma	1.492	0.823-2.704	0.187
Grade of differentiation			<0.001
Well/Moderate	1	reference	
Poor/Undifferentiated	1.207	1.095-1.329	
Tumor size (cm)	1.022	1.014-1.031	<0.001
LNH			<0.001
<12	1	reference	
≥12	0.723	0.670-0.779	
TNM stage			0.001
IIIA	1	reference	
II	1.195	1.079-1.324	

HR = hazard ratio, CI = confidence interval, LNH = number of lymph nodes harvested.

a Includes Native American, Asian, Pacific Islander and Unknown.

**Figure 2:**
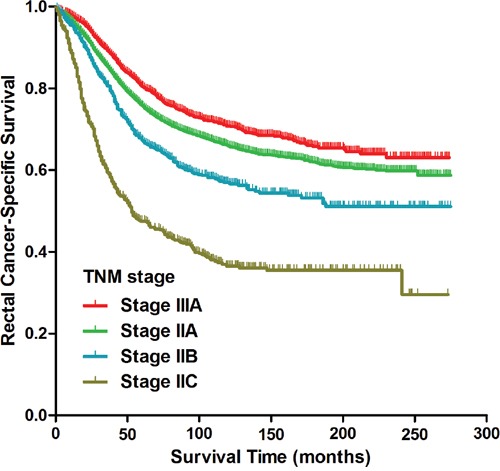
Kaplan-Meier curves of cancer-specific survival for patients with stage IIA, stage IIB, stage IIC and stage IIIA rectal cancer from the SEER database

Patients with stage II rectal cancer was further analyzed as patients with stage IIA, IIB and IIC rectal cancer. Kaplan-Meier analysis showed a significant difference in CSS in patients with stage IIA, IIB, stage IIC and stage IIIA rectal cancer (p<0.001, Figure 2); the 5-year CSS for patients with stage IIA, stage IIB, stage IIC and stage IIIA rectal cancer was 75% (95% CI 75%-75%), 67% (95% CI 66%-67%), 46% (95% CI 45%-46%) and 80% (95% CI 80%-80%), respectively. Multivariate analysis identified TNM stage (p<0.001) as an independent prognostic factor for CSS (Table 4). Compared with patients with stage IIIA rectal cancer, patients with stage IIA rectal cancer (HR 1.113, 95% CI 1.003-1.235, p=0.044), stage IIB rectal cancer (HR 1.493, 95% CI 1.267-1.758, p<0.001) and stage IIC rectal cancer (HR 2.712, 95% CI 2.319-3.171, p<0.001) were more likely to have more unfavorable CSS. Kaplan-Meier curve of overall survival for patients with stage IIA, stage IIB, stage IIC and stage IIIA rectal cancer was shown in Figure S2 (p<0.001).

**Table 4:** Multivariate survival analyses of patients with stage IIA, IIB, IIC and IIIA rectal cancer from the SEER database

Variable	Multivariate analysis		P value
	HR	95% CI	
Age at diagnosis (yrs)			<0.001
≤70	1	reference	
>70	1.691	1.577-1.814	
Race			<0.001
White	1	reference	
Black	1.402	1.240-1.585	<0.001
Other ^a^	0.753	0.659-0.861	<0.001
Histological Type			0.092
Adenocarcinoma	1	reference	
Mucinous adenocarcinoma	1.144	1.009-1.298	0.036
Signet-ring cell carcinoma	1.228	0.677-2.227	0.500
Grade of differentiation			<0.001
Well/Moderate	1	reference	
Poor/Undifferentiated	1.201	1.090-1.324	
Tumor size (cm)	1.018	1.009-1.028	<0.001
LNH			<0.001
<12	1	reference	
≥12	0.720	0.668-0.777	
TNM stage			<0.001
IIIA	1	reference	
IIA	1.113	1.003-1.235	0.044
IIB	1.493	1.267-1.758	<0.001
IIC	2.712	2.319-3.171	<0.001

HR = hazard ratio, CI = confidence interval, LNH = number of lymph nodes harvested.

a Includes Native American, Asian, Pacific Islander and Unknown.

## DISCUSSION

The criterion that differentiates stage III disease from stage II disease is the presence or absence of lymph node metastasis based on the AJCC cancer staging system for rectal cancer. Because of the contradictory oncologic features of stage IIIA rectal cancer, including shallow intestinal wall invasion (T1/T2) and early regional lymph node metastasis, patients with stage IIIA rectal cancer may theoretically exhibit conflicting survival outcomes. On one hand, the disease possesses a relatively limited depth of intestinal wall invasion and metastasis is confined to regional lymph nodes around the primary tumor. Disease in this stage should be easily resected with curative intent and is generally regarded as exhibiting a favorable prognosis. On the other hand, early metastasis to regional lymph nodes with a limited intestinal wall invasion may suggest the aggressive nature of these lesions, which may result in a decreased prognosis [7]. Actually, increased oncologic outcomes have been observed in stage IIIA colorectal cancer compared with the other subgroups of stage III disease, and Mukai et al.[19] suggested that the T1/2N1 category of colorectal cancer should be redefined as stage I or stage II colorectal cancer.

The results from our study suggested that patients with stage IIIA rectal cancer had more favorable CSS than patients with stage II rectal cancer. However, the hazard ratio (1.113) between stage IIA and stage IIIA rectal cancer is quite small, therefore, the clinical significance of survival paradox between stage IIA and stage IIIA rectal cancer may be limited. In surgical procedures of colorectal cancer, every effort should be done to achieve a negative margin. Chu et al.[20] indicated that for patients with stage IIIA colon cancer, only 1% had residual tumor compared with 19% for patients with stage IIB/C colon cancer (p<0.0001), the positive surgical margins may contribute to the survival contradiction between patients with stage IIB/C and stage IIIA colon cancer. The results from our study revealed that patients with stage IIIA rectal cancer had smaller tumor size than patients with stage II disease. For a T1/T2 tumor with small tumor size (i.e., stage IIIA), *en bloc* resection of the primary lesion should be easier to be accomplished than for a T3/T4 tumor with larger tumor size (i.e., stage II) to achieve negative margins status. In addition, surgical proficiency of the operation on locally advanced rectal cancer may vary among different surgeons, leading to a greater rate of positive margin in stage II rectal cancer than in stage IIIA disease [20]. The positive surgical margins may explain the survival paradox between stage IIIA and stage II rectal cancer.

The obvious defect of the current AJCC cancer staging system in colorectal cancer is the relatively over-estimated weighting of node metastasis (N stage) [10, 21]. In malignancies such as esophageal, gastric, breast and lung cancer, stage II tumors are defined by both T category (T1/2) and N category (N1), according to the AJCC cancer staging system. However, in colorectal cancer, apart from patients with distal metastasis (stage IV), all node-positive patients are categorized as stage III regardless of their T status based on the TNM classification [5]. Although lymph node status has been identified as an essential prognostic factor in colorectal cancer that can guide adjuvant therapy and evaluate prognosis [22, 23], the integration of TN categories into a cancer staging system remains complicated. Therefore, the over-estimated weighting of node metastasis in the current AJCC cancer staging system leads to poor monotonicity of gradients from the early to the advanced cancer stages in colorectal cancer [24, 25]. Some researchers have proposed an improved AJCC cancer staging system, in which stage IIIA disease is reclassified as stage I or stage II based on a cluster analysis of TN scores in colorectal cancer [24]. The favorable oncological outcomes of stage IIIA rectal cancer indicate that weighting of the T status in the TNM staging system may be under-estimated, and conventional notions should be reconsidered according to contemporary survival data in rectal cancer.

According to the National Comprehensive Cancer Network (NCCN) guidelines for rectal cancer, preoperative CRT has been recommended as a crucial therapeutic strategy for locally advanced rectal cancer. Growing evidence suggests that patients with locally advanced rectal cancer benefit from preoperative CRT in terms of pCR, local disease control and sphincter preservation in comparison with surgery alone or postoperative CRT [13, 26–28]. However, it's still controversial whether preoperative CRT results in sufficient benefits for low-risk patients with stage IIA rectal cancer to outweigh the toxicity, complication, and inconvenience of treatment [29]. Despite this controversy, findings from the current study have led us to recommend preoperative CRT for patients with stage IIA rectal cancer. For patients with locally advanced rectal cancer with medical contraindication to multimodality therapy, adjuvant therapy following surgery is necessary. The Gastrointestinal Tumor Study Group and National Surgical Adjuvant Breast and Bowel Project Trials demonstrated that for patients with Duke B and Duke C rectal cancer, adjuvant CRT could reduce local recurrence rate and prolong disease-free survival [30, 31]. Therefore, the 1990 National Institute of Health consensus statement recommended chemotherapy combined with radiotherapy as the standard adjuvant therapeutic strategy for pT3 or pN1-2 rectal cancer [17]. However, Gunderson et al.[32] showed that for patients with intermediate-risk stage of rectal cancer (T1-2N1, T3N0), adjuvant CRT could not prolong disease-free survival and overall survival compared with adjuvant chemotherapy alone. The use of CRT following surgery in all intermediate-risk stage of rectal cancer may be excessive. Risk factors such as tumor location, grade of differentiation, circumferential resection margin, lymphovascular invasion and perineural invasion need to be evaluated for individualized treatment, adjuvant chemotherapy alone may be considered for low-risk patients with T1-2N1/T3N0 rectal cancer.

To the best of our knowledge, this is the first comprehensive study to reveal favorable prognosis in and distinctive clinicopathological features of patients with stage IIIA rectal cancer. We used the SEER database to ensure a large sample size, and to be specific, our study included of 12,036 total patients, which guaranteed our findings would have adequate power. However, there are still several limitations to our study. One remarkable limitation of the SEER database is that it does not include record of some important patient- and disease-related variables, including lymphovascular or perineural invasion, comorbidities, intestinal obstruction or penetration, circumferential resection margin, and data on adjuvant chemotherapy. These clinicopathological data may be valuable additions to the current study. Another limitation of our study is that only 34.0% rectal cancer patients had adequate lymph node harvest (≥12), for the reason that patients extracted from the SEER database were diagnosed between 1988 and 2003. The definition of tumor deposits (TDs) has changed in the 5th, 6th, and 7th edition of TNM staging system [3–5]. Nagtegaal et al.[33] revealed that every change in edition of TNM staging system led to a stage migration of between 33% and 64% in patients with TDs. Therefore, the changes in the definition of TDs may be a potential limitation in the accuracy of the SEER data. Because information on the clinical staging of rectal cancer patients treated with preoperative radiotherapy is not available, we are unable to compare the survival difference between patients with clinical stage II rectal cancer and clinical stage IIIA rectal cancer treated with preoperative radiotherapy, therefore, our findings may not be generalizable to these patients.

In conclusion, our results provide the first evidence that patients with stage IIIA disease had more favorable survival outcomes and smaller tumor size compared with patients with stage II rectal cancer. Our findings on the favorable prognosis in and distinctive clinicopathological features of patients with stage IIIA rectal cancer are expected to be validated in other institutions.

## MATERIALS AND METHODS

### Patient selection

The SEER database contains 18 cancer registries covering 26% of the U.S. population and collects and supplies cancer incidence and survival data. We extracted demographic and pathological data for invasive rectal cancer patients between January 1988 and December 2003 from the SEER database (April 2013 release). Patients meeting the following criteria were included in the current analysis: (1) adenocarcinoma, mucinous adenocarcinoma or signet-ring carcinoma of the rectum; (2) known invasion depth and lymph node status; (3) AJCC stage II or stage IIIA; (4) rectal cancer surgically resected with pathology specimen; (5) pathologically confirmed diagnosis; and (6) known survival time and cause of death. Patients were excluded if (1) they received preoperative radiotherapy, (2) they underwent only local tumor excision, (3) their rectal cancer was diagnosed by death certificate or autopsy, or (4) there were other concurrent malignancies. The Fudan University Shanghai Cancer Center Ethical Committee and Institutional Review Board reviewed and approved this study protocol.

### Outcome measures

Records on the following clinicopathological variables were extracted from the SEER database: gender; race; age at and year of diagnosis; primary tumor site; histological type; American Joint Committee on Cancer (AJCC) TNM stage; number of lymph nodes harvested, with 12 as the cutoff value; number of metastatic lymph nodes; depth of intestinal wall invasion; tumor size; grade of differentiation; radiation sequence with surgery; number of primaries; follow-up duration; and SEER classification of cause-specific death. All patients were restaged based on the AJCC cancer stages (7th edition), in which stage IIA was defined as T3N0M0, stage IIB as T4aN0M0, stage IIC as T4bN0M0 and stage IIIA as T1-2N1M0 or T1N2aM0 in rectal cancer. Cancer-specific survival (CSS) from the time of diagnosis to the time of rectal cancer-specific death was the primary outcome. Patients who died from other causes or who were alive at the last follow-up were censored.

### Statistical analysis

Stage II and stage IIIA rectal cancer patient data were summarized using cross-tabulation, and distributions were compared using chi-squared tests. Survival curves were plotted using the Kaplan-Meier method. The log-rank test was used for univariate analysis and variables with a P value less than 0.1 were entered into the multivariate analysis. Multivariate Cox regression analyses were utilized to generate adjusted hazard ratios (HR) and their corresponding 95% confidence intervals (CIs). Tumor size was analyzed as a continuous variable in the multivariate Cox regression analyses. A two-sided P value of less than 0.05 was accepted as statistically significant. All statistical analyses were performed using SPSS statistical program version 20.0 (SPSS Inc., Chicago, IL, USA).

## SUPPLEMENTARY FIGURES


