# Supplementary material for: The survival and clinicopathological differences between patients with stage IIIA and stage II rectal cancer: An analysis of 12,036 patients in the SEER database
Source: Oncotarget. 2016 Oct 28;7(48):79787–96. doi: 10.18632/oncotarget.12970 (PMC5346750; doi:10.18632/oncotarget.12970)
Supplement: Supplementary file 1 [file oncotarget-07-79787-s001.pdf]

The survival and clinicopathological differences between patients with stage IIIA and stage II rectal cancer: An analysis of 12,036 patients in the SEER database

SUPPLEMENTARY FIGURES

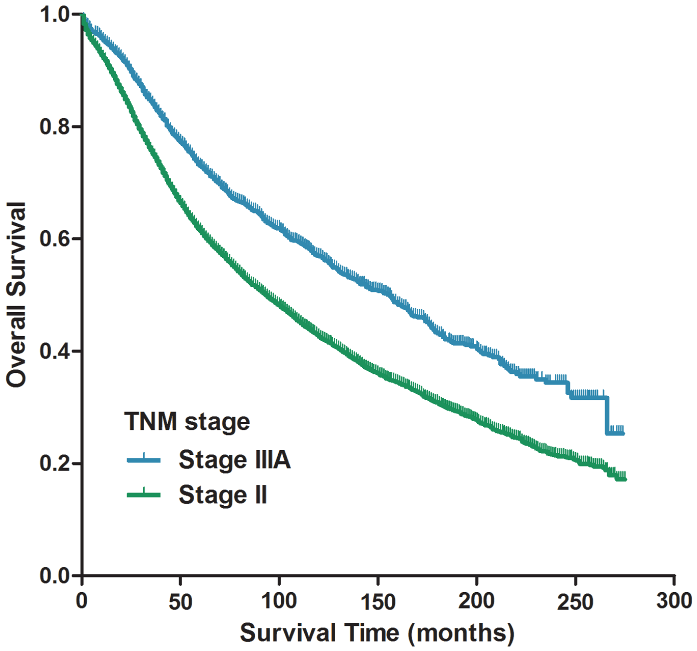

Supplementary Figure S1: Kaplan-Meier curve of overall survival for patients with stage II and stage IIIA rectal cancer from the SEER database.

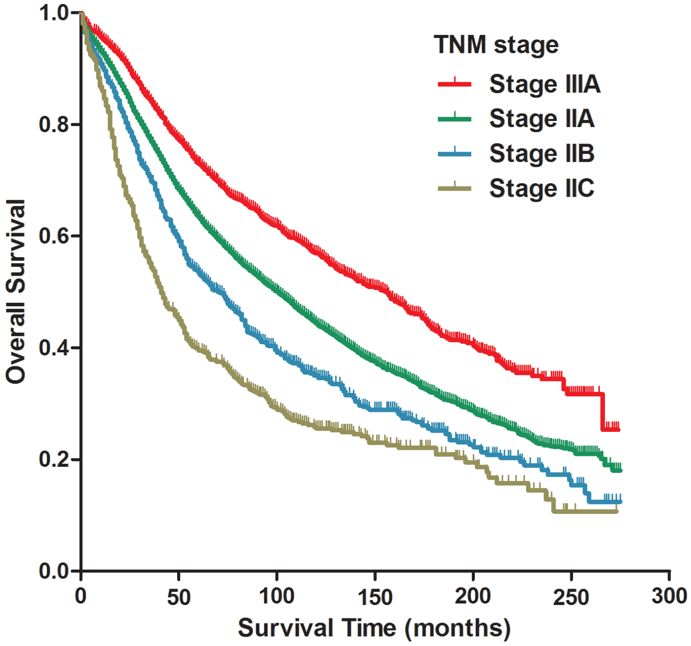

Supplementary Figure S2: Kaplan-Meier curve of overall survival for patients with stage IIA, stage IIB, stage IIC and stage IIIA rectal cancer from the SEER database.
